# Supplementary material for: Determining responsiveness and meaningful changes for the Musculoskeletal Health Questionnaire (MSK-HQ) for use across musculoskeletal care pathways
Source: BMJ Open. 2019 Oct 3;9(10):e025357. doi: 10.1136/bmjopen-2018-025357 (PMC6797397; doi:10.1136/bmjopen-2018-025357)
Supplement: Author's manuscript [file bmjopen-2018-025357.draft_revisions.pdf]

BMJ Open is committed to open peer review. As part of this commitment we make the peer review history of every article we publish publicly available.

When an article is published we post the peer reviewers' comments and the authors' responses online. We also post the versions of the paper that were used during peer review. These are the versions that the peer review comments apply to.

The versions of the paper that follow are the versions that were submitted during the peer review process. They are not the versions of record or the final published versions. They should not be cited or distributed as the published version of this manuscript.

BMJ Open is an open access journal and the full, final, typeset and author-corrected version of record of the manuscript is available on our site with no access controls, subscription charges or pay-per-view fees (http://bmjopen.bmj.com).

If you have any questions on BMJ Open's open peer review process please email info.bmjopen@bmj.com

BMJ Open

Determining Responsiveness and Meaningful Changes for the Musculoskeletal Health Questionnaire (MSK-HQ) for use across musculoskeletal care pathways.

Journal:	*BMJ Open*
Manuscript ID	bmjopen-2018-025357
Article Type:	Research
Date Submitted by the Author:	01-Aug-2018

Complete List of Authors:	Price, Andrew; University of Oxford, Nuffield Department of Orthopaedics, Rheumatology and Musculoskeletal Sciences<br>Ogollah, Reuben; University of Oxford, Nuffield Department of Population Health<br>Kang, Sujin; University of Oxford, Nuffield Department of Orthopaedics, Rheumatology and Musculoskeletal Science<br>Hay, Elaine; Keele University, Institute of primary care and health sciences<br>Barker, Karen L.; University of Oxford, Nuffield Department of Orthopaedics, Rheumatology and Musculoskeletal Sciences<br>Benadetto, Elena; Institute of Musculoskeletal Sciences<br>Smith, Stephanie; Institute of Musculoskeletal Sciences, NDORMs<br>Smith, James; University of Oxford, Nuffield Department of Orthopaedics, Rheumatology and Musculoskeletal Sciences<br>Galloway, James B; Kings College London<br>Ellis, Benjamin; Arthritis Research UK,<br>Rees, Jonathan; University of Oxford,<br>Glyn-Jones, Sion; University of Oxford, Nuffield Department of Orthopaedics, Rheumatology and Musculoskeletal Sciences<br>Beard, David; University of Oxford, Nuffield Dept of Orthopaedics, Rheumatology and Musculoskeletal Sciences<br>Fitzpatrick, Ray; University of Oxford, Nuffield Department of Population Health<br>Hill, Jonathan; Keele University, Institute of primary care and health sciences

Keywords:	musculoskeletal, patient reported outcome, MSK-HQ, responsiveness, minimally important change

SCHOLARONE™
Manuscripts

Determining Responsiveness and Meaningful Changes for the Musculoskeletal Health Questionnaire (MSK-HQ) for use across musculoskeletal care pathways.

Andrew Price[1]

Reuben Ogollah [2]

Sujin Kang[1]

Elaine M Hay [2]

Karen Barker[1]

Elena Benedetto[1]

Stephanie Smith[1]

James Smith[1]

James Gallaway[4]

Benjamin Ellis[5]

Jonathan Rees[1]

Sion Glyn-Jones[1]

David Beard[1]

Ray Fitzpatrick[3]

Jonathan Hill[2]

[1]Nuffield Department of Orthopaedics, rheumatology and Musculoskeletal Science University of Oxford, Nuffield Orthopaedic Centre, Headington, Oxford, OX3 7LD

[2]Arthritis Research UK Primary Care Centre, Research Institute for Primary Care & Health Sciences, Keele University, Keele, ST5 5BG, United Kingdom

[3]Nuffield Department of Population Health, University of Oxford, Oxford, UK

[4] Academic Rheumatology, Faculty of Life Sciences and Medicine, King's College London, London, United Kingdom

[5]Arthritis Research UK, London, UK

Corresponding author of this report:

Professor Andrew Price (PI)

Botnar Research Centre, Nuffield Orthopaedic Centre, Headington, Oxford, OX3 7LD.

andrew.price@ndorms.ox.ac.uk

For peer review only

Word count: 2013

Key words:

Musculoskeletal, patient reported outcome measure, responsiveness, and minimally important change.

For peer review only

For peer review only - http://bmjopen.bmj.com/site/about/guidelines.xhtml

Abstract

Objectives

We have previously developed and validated the Arthritis Research UK Musculoskeletal Health Questionnaire (MSK-HQ) for use across musculoskeletal care pathways, showing encouraging psychometric test results. The objective of this study was to determine the responsiveness of MSK-HQ following MSK treatments and to determine the minimally important change (MIC).

Setting

We collected data in four cohorts from community physiotherapy and secondary-care orthopaedic hip, knee and shoulder clinics.

Participants

610 individuals were recruited; 210 patients treated with physiotherapy for a range of MSK conditions in primary-care; 150 patients undergoing hip replacement, 150 patients undergoing knee replacement and 100 undergoing shoulder surgery in secondary-care.

Outcome measures

Pre-operative data was collected including the MSK-HQ, EQ-5D and the Oxford hip, knee or shoulder score in each joint specific group. The same scores, together with anchor questions were collected post-intervention at 3 months for the physiotherapy group and 6 months for all others. Following COSMIN guidelines responsiveness was assessed using correlation between scores and the MIC was calculated for the entire cohort using receiver operating characteristic curve analysis.

Results

The MSK-HQ demonstrated strong correlation (R=0.73) with EQ-5D across the entire cohort and with each of the joint specific Oxford scores (hip R = 0.87, knee R = 0.92 and shoulder R = 0.77). Moderate correlation was seen between MSK-HQ and EQ-5D across each individual group (R value range 0.60-0.68), apart from the hip group where correlation was strong (R=0.77). The effect size with MSK-HQ was 0.93, in the entire cohort, double that measured

For peer review only - http://bmjopen.bmj.com/site/about/guidelines.xhtml

with EQ-5D (0.43). In all sub-groups MSK-HQ measured a greater treatment effect compared to EQ-5D. The MIC is 5.5 (95% CI 2.7 – 8.3).

Conclusions

Our study demonstrates that the MSK-HQ questionnaire is responsive to change across a range of musculoskeletal conditions, supporting its use as a generic MSK measurement instrument.

For peer review only

Strengths and limitations of this study

- The Musculoskeletal Health Questionnaire (MSK-HQ) has been shown to be responsive across a range of musculoskeletal (MSK) conditions.

- The MSK-HQ performed well in comparison to other quality of life based questionnaires, including the EQ-5D score.

- The Minimally Important Change (MIC) value for the MSK-HQ in the general MSK population has been calculated.

- A limitation of the study is that the MSK-HQ has not been tested across all MSK conditions.

For peer review only

Introduction

Musculoskeletal health problems affect 1 in 4 of the UK adult population and managing this diverse spectrum of conditions accounts for approximately £5 billion of NHS spending annually[1]. Patients with musculoskeletal pain usually enter the clinical pathway via their General Practitioner (GP), presenting with a wide range of different diagnoses and representing up to 14% of all GP consultations [2]. The pathway for providing MSK services in the UK has developed significantly over the last 15 years[3]. Primary care treatment is effective in managing many MSK problems but a significant proportion of patients will be referred through a intermediate care pathway where more diagnostic tests may be undertaken and physiotherapy care provided[4]. A proportion of these patients have symptoms resistant to initial therapy and are referred on to hospital based secondary care for assessment by Orthopaedic, Rheumatology or Sports Medicine doctors[5]. These pathways have become formally established within the UK with the development of integrated MSK clinical assessment and treatment services[6]. The development of care pathways in MSK services has occurred in parallel with the drive towards validated outcome-based assessment of the care provided and value based commissioning[7]. To facilitate this there has been a requirement to create a relevant generic MSK patient reported outcome measure that could be used to assess all MSK patients as they initially enter and then progress through the pathway, regardless of diagnosis[8]. The EQ-5D measurement questionnaire has been previously widely used for this role in MSK care, but some concerns have been raised regarding its lack of responsiveness in this role [9,10].

To address this need the Arthritis Research UK Musculoskeletal Health Questionnaire (MSK-HQ) was developed[8]. This is a generic musculoskeletal (MSK) patient reported outcome measure, designed to be used across the full spectrum of MSK conditions. The MSK-HQ contains 14 items capturing symptoms and functional problems that patients with MSK conditions have identified as important. These include: pain severity, physical function, work interference, social interference, sleep, fatigue, emotional health, physical activity, independence, understanding, confidence to self-manage, and overall-impact. The MSK-HQ has been shown to have encouraging psychometric test results with high completion rates

For peer review only

and excellent test-retest reliability. The score demonstrates very good convergent validity when measured against reference standards[8].

The specific aim of this study was to determine the responsiveness of the MSK-HQ in measuring clinical change following treatments, across different MSK pathways. In addition the study aimed to determine the minimally important change for MSK-HQ.

For peer review only

Methods

Cohorts:

We have developed a series of cohorts of patients to validate the MSK-HQ as previously described. [8] We used follow-up outcome data from the cohorts to determine the MSK-HQ's responsiveness and minimally important change.

Community physiotherapy cohort:

Patients were recruited from community musculoskeletal physiotherapy clinics in five UK West Midlands towns (Middlewich, Congleton, Wombourne, Cheadle, and Wolverhampton), where NHS treatment is offered after referral by the GP. Patients were recruited directly in the clinic and the inclusion criteria were age >18 and referral from a GP for physiotherapy treatment. No further inclusion/exclusion criteria were used, creating a study group with a heterogeneous range of unspecified musculoskeletal problems. Patients agreed to fill out questionnaires pre-treatment and at 3-months after first consultation.

Secondary care Orthopaedic cohorts (knee, hip, shoulder):

From the Nuffield Orthopaedic Centre in Oxford we recruited separate knee, hip and shoulder cohorts from patients who had been listed for surgery. Inclusion criteria were age >18 and patient listed for (1) knee replacement surgery, (2) hip replacement surgery of (3) shoulder surgery including open and arthroscopic procedures (excluding instability surgery). Patients were asked to fill out a pre-operative questionnaire and again at 6-month post-surgery. The shoulder cohort had been expanded to 100 compared to the 60 previously reported [8].

Outcome measures

All participants were asked to complete an MSK-HQ and EQ-5D at baseline and final follow-up[11]. In addition the Orthopaedic Cohorts filled out a joint specific patient reported outcome measure; Oxford Knee Score (OKS), Oxford Hip Score (OHS) or an Oxford Shoulder Score (OSS). In each group the same scores were recorded at final follow-up (3 months for physiotherapy and 6 months for orthopaedic cohorts)[12–14].

For peer review only - http://bmjopen.bmj.com/site/about/guidelines.xhtml

When reporting the MSK-HQ all 14 items are summed together (responses coded from ‘not at all’ = 4 to ‘extremely’ = 0, except for items 12 and 13 which have the response options in the reverse order) providing a range from 0-56, with higher scores indicating better MSK health status[8].

Evaluation of Responsiveness and Calculation of Minimally Important Change

We followed COSMON guidelines for evaluation of responsiveness and calculation of minimally important change[15].

For each of the prospectively collected cohorts we compared the baseline and follow-up scores (3 months for the Physiotherapy cohort and 6 months for Orthopaedic cohorts). Firstly we examined the magnitude and direction of change calculating correlation coefficients for comparison of MSK-HQ and other measures, within the different groups.
We then performed sub-group analysis calculating and comparing the standardised effect size (SES - the mean change score divided by the standard deviation of the measure at baseline) and the standardised response mean (SRM – the mean change score divided by the standard deviation of the change scores) for the outcome measure used within each cohorts collected.

For the entire cohort the clinically relevant change (improvement or deterioration) was determined by calculating the minimally important change (MIC) using receiver operating characteristic (ROC) curve analysis. For the external anchor we used the following transition item from the Outcome and Experience Questionnaire to identify change in clinical state compared to baseline (Question: ‘How would you now rate the problem you recently came to clinic or hospital for?’ Response: ‘Much better’, ‘a little better’, ‘the same’, ‘a little worse’ or ‘much worse’)[16]. For calculation of the minimally important change the responses were dichotomized using two response groups; (1) improvement = a little better and (2) no improvement = same.

For peer review only

Patient and public involvement

The MSK-HQ questionnaire in a patient reported outcome measure and its development has involved patients in each of the following stages; development of the research question, development of the outcome measures itself and participating in the research.

For peer review only

Results

Demographic data

There were 610 patients in total who consented to participate in the four cohort studies (210 physiotherapy patients, 150 hip, 150 knee, 100 shoulder). Baseline population characteristics for the overall sample and by each cohort are summarised in Table 1, showing a mean age of 56 years (SD 16.9) and with a split of 56.5% female and 43.5% male. As expected the symptom episode duration was much greater in the secondary care cohorts compared to the community physiotherapy cohort.

Descriptive analysis of baseline and follow-up scores.

Table 2 demonstrates that across the entire cohort there was an improvement in mean baseline score to follow-up score for both MSK-HQ and EQ-5D. A very similar pattern was seen for the physiotherapy cohort. In the joint specific cohorts the same trend occurred across all three scores used in each setting. The response rate for the entire cohort was 70%.

Responsiveness

There was a strong correlation between change scores for MSK-HQ and non-disease specific EQ-5D across the entire cohort with an R value of 0.73 (Table 3). There was moderate correlation between the same measures in the physiotherapy (0.67), knee (0.67) and shoulder cohorts (0.57), with strong correlation in the hip cohort (0.72). When comparing change scores between MSK-HQ and Oxford scores in the joint specific cohorts the correlations for OKS (0.93) and OHS (0.82) were particularly strong, with moderate correlation seen with the OSS (0.61).

Across all cohorts the MSK-HQ measured a relatively large mean effect size for treatment ranging between 2.14 in the hip cohort and 0.93 in the Physio cohort. Within the Physio cohort greater responsiveness was shown with MSK-HQ compared to EQ-5D, with over twice the effect size measured at 3 months. In the knee cohort MSK-HQ measured a similar effect size to the OKS (1.53 and 1.52 respectively) at 6 months, both of which were greater than that calculated using EQ-5D (0.94). A similar pattern was seen within the hip cohort.

For peer review only - http://bmjopen.bmj.com/site/about/guidelines.xhtml

Within the shoulder cohort the pattern was different with MSK-HQ measuring a greater effect size than OSS and EQ-5D, which delivered similar results across the cohort (both 0.73). The findings were supported by the calculated standardized response mean values (Table 4).

Minimally Important Change

The MIC for MSK-HQ was 5.5 (95% CI 2.7 – 8.3) based on the optimal cut-off value with specificity 0.66 and sensitivity 0.65, with area under the ROC curve 0.66 (See Figure 1).

For peer review only

Discussion

The results of this study demonstrate that the MSK-HQ PROM is responsive to change across patients with a range of musculoskeletal conditions. When considering change scores across the entire cohort the MSK-HQ performed well compared to EQ-5D with a strong correlation between the scores. Within the subgroups the MSK-HQ correlated very well with change measured using the Oxford hip, knee and shoulder scores. This was supported by measured effect sizes, particularly for OKS and OHS. For the community physiotherapy group the correlation with EQ-5D was moderate but there was a much larger measured effect size using the MSH-HQ. The trend for the new score to measure a larger effect size when compared to EQ-5D was seen across all the cohorts.

The requirement to integrate PROMs into clinical management and outcome based commissioning in primary and intermediate care has expanded[17]. The MSK-HQ PROM was designed to be applicable across MSK conditions and to be meaningful to patients particularly in the primary and intermediate care setting[8]. Therefore it is important to reflect on the results of the MSK-HQ across the whole mixed cohort and the community physiotherapy cohort, which does represent a truly mixed population of different MSK conditions. Previous work has shown that the MSK-HQ has excellent test-retest reliability and strong convergent validity with reference standards [8]. Results from the entire cohort and the physiotherapy cohort suggest that the MSK-HQ is responsive to change in the general MSK population and is applicable to the primary and intermediate care setting. Considering its use in this setting we have calculated a single minimally important change (MIC) value for the MSK-HQ for application across all MSK conditions. The MIC value represents the smallest change in MSK-HQ score that represents a meaningful change in symptoms over time for an individual patient within a care pathway, a score that has been calculated for many other outcome measures.[18] Physiotherapy has a very important role to play in the initial non-operative management of nearly all MSK conditions and for many years the outcome measure of choice in this clinical space has been EQ-5D [19–24]. Encouragingly he MSK-HQ performed favorably in comparison to EQ-5D in terms of responsiveness to change across the cohort.

For peer review only - http://bmjopen.bmj.com/site/about/guidelines.xhtml

Despite the MSH-HQ not being joint or condition specific the new score performed well in responsiveness when compared to the Oxford hip, knee and shoulder scores. In particular the score appears to measure change in response to hip and knee arthroplasty in a similar manner to the OKS and OHS with very high measured correlation coefficients and similar effect sizes.

Our study does have missing data from the follow-up cohort. However overall the effective response rate for the entire cohort was 70%, which is considered acceptable[25]. Although the study population does not cover all MSK diagnoses we believe it is relatively representative of a cohort of patients who would be assessed in primary or intermediate care.

The expansion of MSK intermediate care in within the NHS has highlighted the need for a generic PROM to assist in developing outcome based commissioning of care. The measurement property profile of the MSK-HQ PROM appears to support its use in this area, although its role is still evolving. Increasingly there is a common clinical pathway for most MSK patients within the NHS. Patients initially seek advice and treatment from their GP and if their condition does not settle are referred to a musculoskeletal intermediate care referral hub, which acts as a gateway to secondary care. The pathway is not joint or condition specific so a generic PROM to measure clinical change and response to treatment is required for the process. The MSK-HQ appears to demonstrate the appropriate measurement properties and responsiveness to fulfill this role.

Conclusion

In summary the findings from this study show that the MSK-HQ appears to be responsive to change across a range of MSK conditions, performing favourably when compared to EQ-5D and joint specific scores.

For peer review only

References:

1. Margham T. Musculoskeletal disorders: time for joint action in primary caree. Br J Gen Pract. 2010;61(592):657–8.

2. Jordan KP, Kadam UT, Hayward R, Porcheret M, Young C, Croft P. Annual consultation prevalence of regional musculoskeletal problems in primary care : an observational study. 2010;

3. Maddison P, Jones J, Breslin A, Barton C, Fleur J, Lewis R, et al. Quality improvement report Improved access and targeting of musculoskeletal services. 2004;1325–7.

4. Hussenbux A, Morrissey D, Joseph C, Mcclellan CM. Intermediate Care pathways for musculoskeletal conditions – Are they working ? A systematic review. Physiotherapy [Internet]. 2015;101(1):13–24. Available from: http://dx.doi.org/10.1016/j.physio.2014.08.004

5. Jl J, Ma H, Eej M, Ne F. Interventions to improve adherence to exercise for chronic musculoskeletal pain in adults (Review). 2010;(1).

6. Roberts C, Dolman EA, Adebajo AO, Underwood M. A national qualitative survey of community- based musculoskeletal services in the UK. 2003;(May):1074–8.

7. DH. The Musculoskeletal Services Framework A joint responsibility : doing it differently. [Internet]. 2006 [cited 2018 Apr 14]. Available from: www.dh.gov.uk/publications

8. Hill JC, Kang S, Benedetto E, Myers H, Blackburn S, Smith S, et al. Development and initial cohort validation of the Arthritis Research UK Musculoskeletal Health Questionnaire (MSK-HQ) for use across musculoskeletal care pathways. BMJ Open. 2016;6(8):1–10.

9. Whynes DK, McCahon RA, Ravenscroft A, Hodgkinson V, Evley R, Hardman JG. Responsiveness of the EQ-5D health-related quality-of-life instrument in assessing low back pain. Value Heal [Internet]. 2013;16(1):124–32. Available from: http://dx.doi.org/10.1016/j.jval.2012.09.003

10. Tordrup D, Mossman J, Kanavos P. Responsiveness of the EQ-5D to clinical change: Is the patient experience adequately represented? Int J Technol Assess Health Care. 2014;30(1):10–9.

11. Lloyd MHCGA. Development and preliminary testing of the new five-level version of EQ-5D (EQ-5D-5L). 2011;1727–36.

12. Dawson J, Fitzpatrick R, Carr A. Questionnaire on the perceptions of patients about shoulder surgery. J Bone Joint Surg Br [Internet]. 1996;78(4):593–600. Available

For peer review only - http://bmjopen.bmj.com/site/about/guidelines.xhtml

from: http://www.ncbi.nlm.nih.gov/pubmed/8682827

13. Dawson J, Fitzpatrick R, Carr A, Murray D. Questionnaire on the Perceptions of Patients About Total Hip Replacement. J Bone Jt Surg Br [Internet]. 2015;78–B(2):185–90. Available from: http://www.bjj.boneandjoint.org.uk/content/78-B/2/185.full.pdf+html

14. Dawson J, Fitzpatrick R, Murray D, Carr A. Questionnaire on the Perceptions of Patients about Total Knee Replacement. J Bone Joint Surg Br [Internet]. 1998;80(1):63–9. Available from: http://www.ncbi.nlm.nih.gov/entrez/query.fcgi?cmd=Retrieve&db=PubMed&dopt=Citation&list_uids=9460955

15. COSMIN. COSMIN checklist manual [Internet]. 2012 [cited 2018 Jun 3]. p. 56. Available from: http://www.cosmin.nl/images/upload/files/COSMIN checklist manual v9.pdf

16. Gibbons E, Hewitson P, Morley D, Jenkinson C, Fitzpatrick R. The Outcomes and Experiences Questionnaire: development and validation. Patient Relat Outcome Meas [Internet]. 2015;6:179–89. Available from: http://www.ncbi.nlm.nih.gov/pubmed/26213480%5Cnhttp://www.pubmedcentral.nih.gov/articlerender.fcgi?artid=PMC4509457

17. The Musculoskeletal Services Framework A joint responsibility : doing it differently.

18. Beard DJ, Harris K, Dawson J, Doll H, Murray DW, Carr AJ, et al. Meaningful changes for the Oxford hip and knee scores after joint replacement surgery. J Clin Epidemiol. 2015;68(1):73–9.

19. Desmeules F, Roy J-S, MacDermid JC, Champagne F, Hinse O, Woodhouse LJ. Advanced practice physiotherapy in patients with musculoskeletal disorders: a systematic review. BMC Musculoskelet Disord [Internet]. 2012;13(1):107. Available from: http://bmcmusculoskeletdisord.biomedcentral.com/articles/10.1186/1471-2474-13-107

20. Holdsworth L, Webster V, McFayden A. What are the costs to NHS Scotland of self-referral to physiotherapy? Physiotherapy. 2007;93:3–11.

21. Holdsworth L, Webster V, McFayden A. What are the costs to NHS Scotland of self-referral to physiotherapy? Physiotherapy. 2007;

22. McGill T. Effectiveness of physical therapists servicing as primary care musculoskeletal providers as compared to family practice providers in a deployed combat

For peer review only

location: a retrospective medical chart review. Mil Med. 2013;178(10):115–1120.

23. Fritz JM, Cleland JA, Brennan GP. Does adherence to the guideline recommendations for active treatments improve quality of care for patients with acute low back pain delivered by physical therapists? Med Care [Internet]. 2007;45(10):973–80. Available from: http://search.proquest.com/docview/621889514?accountid=9941

24. Childs JD, Whitman JM, Sizer PS, Pugia ML, Flynn TW, Delitto A. A description of physical therapists' knowledge in managing musculoskeletal conditions. BMC Musculoskelet Disord. 2005;6.

25. Fincham JE. Response rates and responsiveness for surveys, standards, and the Journal. Am J Pharm Educ. 2008;72(2):43.

For peer review only

Figure Legends

Figure 1:

A graph showing the receiver operating characteristic (ROC) analysis using MSK-HQ and EQ-5D Index change scores across the entire cohort.

For peer review only

Patient characteristics	*All participants*	*Physio*	*Hip*	*Knee*	*Shoulder*
**Number**	610	210	150	150	100
**Age (years), *mean (SD)***	56.0 *(16.9)*	53.5 *(15.5)*	55.6 *(17.2)*	65.7 *(13.8)*	53.0 *(16.7)*
**Female, *n (%)***	326 *(56.5)*	112 *(54.1)*	88 *(60.7)*	89 *(62.2)*	37 *(45.1)*
**Male, *n (%)***	251 *(43.5)*	95 *(45.9)*	57 *(39.3)*	54 *(37.8)*	45 *(54.9)*
**Episode duration (months), *median (IQR)***	12 *(2.5, 36)*	3.8 *(1.2, 8)*	24 *(10, 60)*	23 *(6, 60)*	21.5 *(6, 48)*

Table 1:

A table showing demographic characteristics of patients recruited across the four cohorts.

For peer review only

Cohort	Surveys for each Cohort	Baseline Mean	Baseline SD	Follow-up Mean	Follow-up SD
**Total**	MSK-HQ [0,56]	28.6	9.6	41.3	11.4
(n=610)	EQ-5D-5L [-0.59,1]	0.5	0.3	0.7	0.2
**Physio**	MSK-HQ	30.5	9.6	40.0	11.3
(n=210)	EQ-5D-5L [-0.59,1]	0.6	0.3	0.7	0.2
**Hip**	MSK-HQ	24.9	8.3	43.0	12.0
(n=150)	EQ-5D-5L [-0.59,1]	0.4	0.2	0.7	0.3
	OHS [0,48]	20.4	8.6	37.4	10.0
**Knee**	MSK-HQ	27.5	9.0	40.8	11.5
(n=150)	EQ-5D-5L [-0.59,1]	0.5	0.3	0.7	0.2
	OKS [0,48]	20.9	8.8	34.6	10.1
**Shoulder**	MSK-HQ	32.1	10.4	42.9	9.4
(n=100)	EQ-5D-5L [-0.59,1]	0.6	0.3	0.7	0.2
	OSS [0,48]	29.6	10.3	37.5	7.4

Missing data for MSK-HQ: all participants n=50 (8.2%), Physio cohort n=5 (2.4%), hip cohort n=4 (2.7%), knee cohort n=22 (14.7%), shoulder cohort n=19 (19%).

Table 2:

A table showing base line and follow-up outcomes scores for each cohort.

For peer review only

Instrument	Comparator		Spearman Correlation	Pearson Correlation
			s=(95% CIs)	r=(95% CIs)
**MSK-HQ**	***EQ-5D***	*Total*	0.73 (0.67 , 0.77)	0.73 (0.67 , 0.77)
**MSK-HQ**	***EQ-5D***	*Physio*	0.67 (0.57 , 0.76)	0.67 (0.56 , 0.76)
		Hip	0.77 (0.68 , 0.84)	0.72 (0.62 , 0.81)
		Knee	0.68 (0.56 , 0.78)	0.67 (0.54 , 0.76)
		Shoulder	0.60 (0.34 , 0.77)	0.57 (0.29 , 0.76)
**MSK-HQ**	***OHS***	*Hip*	0.87 (0.81 , 0.91)	0.82(0.73 , 0.88)
	OKS	*Knee*	0.92 (0.88 , 0.95)	0.93 (0.89 , 0.95)
	OSS	*Shoulder*	0.77 (0.58 , 0.88)	0.61 (0.33 , 0.79)

Table 3:

A table displaying correlations in change score between MSK-HQ and other established outcome score in the entire group and the cohorts (physiotherapy, hip, knee and shoulder).

For peer review only

Cohort		Effect size (95% CIs) [1]	SRM (95% CIs) [2]
**Physio**	MSK-HQ *n=103*	0.93 (0.66,1.20)	0.99 (0.71,1.26)
	EQ-5D-5L *n=102*	0.43 (0.18,0.68)	0.46 (0.21,0.71)
**Knee**	MSK-HQ *n=105*	1.53 (1.18,1.86)	1.05 (0.74,1.35)
	OKS *n=112*	1.52 (1.19,1.85)	1.26 (0.95,1.57)
	EQ-5D-5L *n=113*	0.94 (0.65,1.23)	0.87 (0.58,1.15)
**Hip**	MSK-HQ *n=103*	2.14 (1.73,2.53)	1.56 (1.21,1.90)
	OHS *n=92*	1.93 (1.53,2.33)	1.59 (1.22,1.95)
	EQ-5D-5L *n=102*	1.21 (0.88,1.52)	1.03 (0.72,1.34)
**Shoulder**	MSK-HQ *n=36*	1.05 (0.52,1.57)	1.08 (0.55,1.60)
	OSS *n=34*	0.73 (0.22,1.23)	0.99 (0.45,1.51)
	EQ-5D-5L *n=38*	0.73 (0.24,1.20)	0.76 (0.29,1.24)

1. Using baseline (BL) standard deviation (SD) 2. Using paired differences standard deviation (SD)

w only

Table 4:

A table displaying effect size and standard response mean for MSK-HQ and other established outcome score in the 4 cohorts (physiotherapy, hip, knee and shoulder).

Acknowledgements

The authors thank the clinical teams and patients that participated in the study. The authors acknowledge support from the Oxford NIHR Biomedical Research Centre.

Contributors

AP, RF, BE, DB, EH, JG, JCH contributed to the conception and design of the work .

JCH, EH, HM, SS, SK, EB, JS, KB, DB, JR, SG-J and AP were involved in running the cohorts and collecting data.

RO, AP, SK and JCH were involved in the analyses and AP, JCH and RF in the interpretation of the data.

AP, RO, EH, KB, SS, JS, JG, JR, SG-J, DB, EB, RF, JH, BE were involved in the drafting of the manuscript and its revision for important intellectual content, and gave final approval for the manuscript.

Funding

This project was funded by Arthritis Research UK (Ref. 20518). Professor Andrew Price is part funded through the Oxford NIHR Biomedical Research Centre.

Competing interests

None declared.

Patient consent

All participants provided informed written consent.

Ethics approval

Ethical approval for this study was obtained through the IRAS system (IRAS ref. 168971) via Wales REC 4 committee (REC ref. 15/WA/0040) .

Data sharing statement

Additional data can be accessed on request to the corresponding author: andrew.price@ndorms.ox.ac.uk

For peer review only

For peer review only

For peer review only - http://bmjopen.bmj.com/site/about/guidelines.xhtml

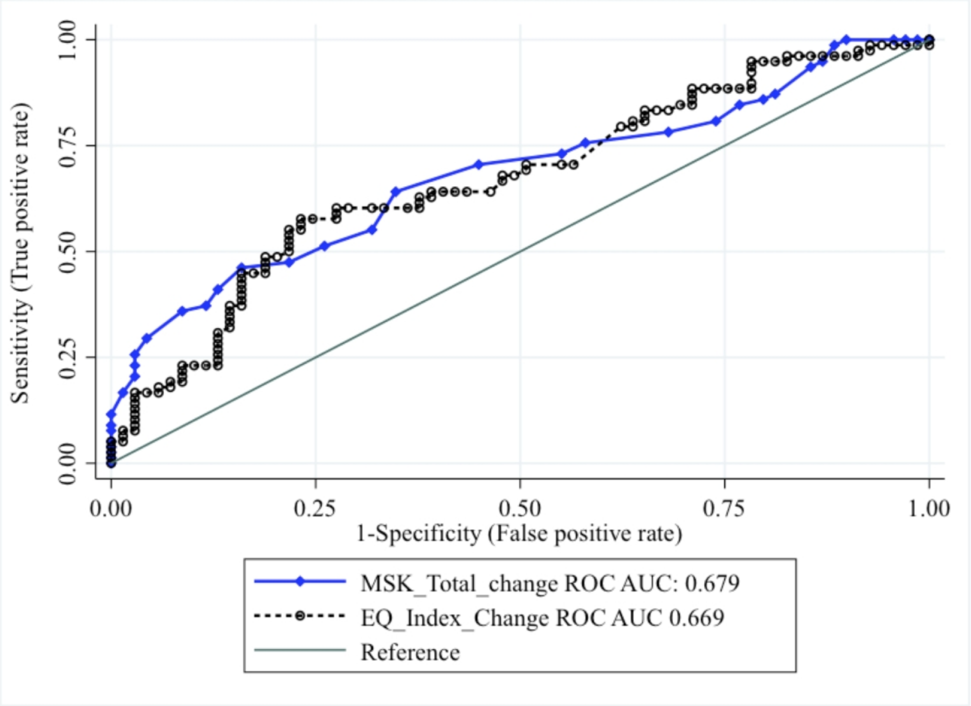

A graph showing the receiver operating characteristic (ROC) analysis using MSK-HQ and EQ-5D Index change scores across the entire cohort.

209x148mm (300 x 300 DPI)

BMJ Open

Determining Responsiveness and Meaningful Changes for the Musculoskeletal Health Questionnaire (MSK-HQ) for use across musculoskeletal care pathways.

Journal:	*BMJ Open*
Manuscript ID	bmjopen-2018-025357.R1
Article Type:	Original research
Date Submitted by the Author:	21-Mar-2019
Complete List of Authors:	Price, Andrew; University of Oxford, Nuffield Department of Orthopaedics, Rheumatology and Musculoskeletal Sciences<br>Ogollah, Reuben; University of Oxford, Nuffield Department of Population Health<br>Kang, Sujin; University of Oxford, Nuffield Department of Orthopaedics, Rheumatology and Musculoskeletal Science<br>Hay, Elaine; Keele University, Institute of primary care and health sciences<br>Barker, Karen L.; University of Oxford, Nuffield Department of Orthopaedics, Rheumatology and Musculoskeletal Sciences<br>Benedetto, Elena; University of Oxford, Nuffield Department of Orthopaedics, Rheumatology and Musculoskeletal Sciences<br>Smith, Stephanie; Institute of Musculoskeletal Sciences, NDORMs<br>Smith, James; University of Oxford, Nuffield Department of Orthopaedics, Rheumatology and Musculoskeletal Sciences<br>Galloway, James B; Kings College London<br>Ellis, Benjamin; Arthritis Research UK,<br>Rees, Jonathan; University of Oxford,<br>Glyn-Jones, Sion; University of Oxford, Nuffield Department of Orthopaedics, Rheumatology and Musculoskeletal Sciences<br>Beard, David; University of Oxford, Nuffield Dept of Orthopaedics, Rheumatology and Musculoskeletal Sciences<br>Fitzpatrick, Ray; University of Oxford, Nuffield Department of Population Health<br>Hill, Jonathan; Keele University, Institute of primary care and health sciences
**Primary Subject Heading**:	Patient-centred medicine
Secondary Subject Heading:	Health services research, Public health
Keywords:	musculoskeletal, patient reported outcome, MSK-HQ, responsiveness, minimally important change, Musculoskeletal disorders < ORTHOPAEDIC & TRAUMA SURGERY

For peer review only - http://bmjopen.bmj.com/site/about/guidelines.xhtml

Determining Responsiveness and Meaningful Changes for the Musculoskeletal Health Questionnaire (MSK-HQ) for use across musculoskeletal care pathways.

Andrew Price[1]

Reuben Ogollah [2]

Sujin Kang[1]

Elaine M Hay [2]

Karen Barker[1]

Elena Benedetto[1]

Stephanie Smith[1]

James Smith[1]

James Galloway[4]

Benjamin Ellis[5]

Jonathan Rees[1]

Sion Glyn-Jones[1]

David Beard[1]

Ray Fitzpatrick[3]

Jonathan Hill[2]

[1]Nuffield Department of Orthopaedics, rheumatology and Musculoskeletal Science University of Oxford, Nuffield Orthopaedic Centre, Headington, Oxford, OX3 7LD

[2]Arthritis Research UK Primary Care Centre, Research Institute for Primary Care & Health Sciences, Keele University, Keele, ST5 5BG, United Kingdom

[3]Nuffield Department of Population Health, University of Oxford, Oxford, UK

[4] Academic Rheumatology, Faculty of Life Sciences and Medicine, King's College London, London, United Kingdom

[5]Arthritis Research UK, London, UK

Corresponding author of this report:

Professor Andrew Price (PI)

Botnar Research Centre, Nuffield Orthopaedic Centre, Headington, Oxford, OX3 7LD.

andrew.price@ndorms.ox.ac.uk

For peer review only

Word count: 2013

Key words:
Musculoskeletal, patient reported outcome measure, responsiveness, and minimally important change.

For peer review only

For peer review only - http://bmjopen.bmj.com/site/about/guidelines.xhtml

Abstract

Objectives

We have previously developed and validated the Arthritis Research UK Musculoskeletal Health Questionnaire (MSK-HQ) for use across musculoskeletal care pathways, showing encouraging psychometric test results. The objective of this study was to determine the responsiveness of MSK-HQ following MSK treatments and to determine the minimally important change (MIC).

Setting

We collected data in four cohorts from community physiotherapy and secondary-care orthopaedic hip, knee and shoulder clinics.

Participants

592 individuals were recruited; 210 patients treated with physiotherapy for a range of MSK conditions in primary-care; 150 patients undergoing hip replacement, 150 patients undergoing knee replacement and 82 undergoing shoulder surgery in secondary-care.

Outcome measures

Pre-operative data was collected including the MSK-HQ, EQ-5D and the Oxford hip, knee or shoulder score in each joint specific group. The same scores, together with anchor questions were collected post-intervention at 3 months for the physiotherapy group and 6 months for all others. Following COSMIN guidelines responsiveness was assessed using correlation between scores and the MIC was calculated for the entire cohort using receiver operating characteristic curve analysis.

Results

The MSK-HQ demonstrated strong correlation (R=0.73) with EQ-5D across the entire cohort and with each of the joint specific Oxford scores (hip R = 0.87, knee R = 0.92 and shoulder R = 0.77). Moderate correlation was seen between MSK-HQ and EQ-5D across each individual group (R value range 0.60-0.68), apart from the hip group where correlation was strong (R=0.77). The effect size with MSK-HQ was 0.93, in the entire cohort, double that measured

For peer review only - http://bmjopen.bmj.com/site/about/guidelines.xhtml

with EQ-5D (0.43). In all sub-groups MSK-HQ measured a greater treatment effect compared to EQ-5D. The MIC is 5.5 (95% CI 2.7 – 8.3).

Conclusions

Our study demonstrates that the MSK-HQ questionnaire is responsive to change across a range of musculoskeletal conditions, supporting its use as a generic MSK measurement instrument.

For peer review only

Strengths and limitations of this study

- The Musculoskeletal Health Questionnaire (MSK-HQ) has been shown to be responsive across a range of musculoskeletal (MSK) conditions.
- The MSK-HQ performed well in comparison to other quality of life based questionnaires, including the EQ-5D score.
- The Minimally Important Change (MIC) value for the MSK-HQ in the general MSK population has been calculated.
- A limitation of the study is that the MSK-HQ has not been tested across all MSK conditions.

For peer review only

Introduction

Musculoskeletal health problems affect 1 in 4 of the adult population and managing this diverse spectrum of conditions is expensive, for instance in the NHS accounting for approximately £5 billion of spending annually[1]. Patients with musculoskeletal pain usually enter the clinical pathway via their primary care doctor presenting with a wide range of different diagnoses and representing up to 14% of all consultations [2]. Given the scale of the MSK healthcare burden new ways of providing care are developing across all healthcare systems. Within the UK the pathway for providing MSK services has developed significantly over the last 15 years[3]. Primary care treatment is effective in managing many MSK problems but a significant proportion of patients will be referred through a intermediate care pathway where more diagnostic tests may be undertaken and physiotherapy care provided[4]. A proportion of these patients have symptoms resistant to initial therapy and are referred on to hospital based secondary care for assessment by Orthopaedic, Rheumatology or Sports Medicine doctors[5]. These pathways have become formally established within the UK with the development of integrated MSK clinical assessment and treatment services[6]. The development of care pathways in MSK services has occurred in parallel with the drive towards validated outcome-based assessment of the care provided and value based commissioning[7]. To facilitate this there has been a requirement to create a relevant generic MSK patient reported outcome measure that could be used to assess all MSK patients as they initially enter and then progress through the pathway, regardless of diagnosis[8]. This requirement reflects the increasing need across all healthcare models to embed the measurement of clinical change over time. To achieve this a responsive patient reported outcome measure is required. The European Quality of Life Questionnaire (EQ-5D-5L) patient measure has been previously widely used for this role in MSK care, but some concerns have been raised regarding its lack of responsiveness[9,10]. This may reflect the measurement properties of EQ-5D-5L as a generic healthcare quality of life measure, rather than a condition or disease specific measure. More specifically the EQ-5D-5L individual items have no musculo-skeletal context for patients which is an important characteristic for embedding them in a clinical pathway.

For peer review only

To address this need the Arthritis Research UK Musculoskeletal Health Questionnaire (MSK-HQ) was developed[8]. This is a generic musculoskeletal (MSK) patient reported outcome measure, designed to be used across the full spectrum of MSK conditions. The MSK-HQ contains 14 items capturing symptoms and functional problems that patients with MSK conditions have identified as important. These include: pain severity, physical function, work interference, social interference, sleep, fatigue, emotional health, physical activity, independence, understanding, confidence to self-manage, and overall-impact. We have previously shown that the MSK-HQ has encouraging psychometric test results with high completion rates (94%), excellent test-retest reliability (Intraclass Correlation Coefficient = 0.84) and a high level of internal consistency (Cronbach's α = 0.88)[8]. In addition the score demonstrates very good convergent validity when measured against EQ-5D with a Spearman's rank correlation coefficient of 0.81, respectively [8].

The specific aim of this study was to determine the responsiveness of the MSK-HQ in measuring clinical change as a single generic MSK measure following treatment across different MSK pathways. In addition the study aimed to determine the minimally important change for MSK-HQ.

For peer review only

Methods

Cohorts:

We have developed a series of cohorts of patients to validate the MSK-HQ as previously described. [8] We used follow-up outcome data from the cohorts to determine the MSK-HQ's responsiveness and minimally important change, with data collected longitudinally at two time points.

Community physiotherapy cohort:

Patients were recruited from community musculoskeletal physiotherapy clinics in five UK West Midlands towns (Middlewich, Congleton, Wombourne, Cheadle, and Wolverhampton), where NHS treatment is offered after referral by the GP. Patients were invited to take part and recruited directly in the clinic having received a study information pack. The inclusion criteria were age >18 and referral from a GP for physiotherapy treatment. No further inclusion/exclusion criteria were used, creating a study group with a heterogeneous range of unspecified musculoskeletal problems. Patients consented to fill out questionnaires pre-treatment and at 3-months after first consultation.

Secondary care Orthopaedic cohorts (knee, hip, shoulder):

From the Nuffield Orthopaedic Centre in Oxford we recruited separate knee, hip and shoulder cohorts from patients who had been listed for surgery. Patients were approached in their pre-assessment clinic appointment prior to surgery having received a study information pack. Inclusion criteria were age >18 and patient listed for (1) knee replacement surgery, (2) hip replacement surgery of (3) shoulder surgery including open and arthroscopic procedures (excluding instability surgery). Patients consented to fill out a pre-operative questionnaire and again at 6-month post-surgery. The shoulder cohort had been expanded to 82 compared to the 60 previously reported [8].

Outcome measures

All participants were asked to complete the MSK-HQ and EQ-5D-5L (the primary reference measure) as baseline and final follow-up as the second time point. .The MSK-HQ is a 14 item questionnaire assessing pain severity, physical function, work interference, social

For peer review only - http://bmjopen.bmj.com/site/about/guidelines.xhtml

interference, sleep, fatigue, emotional health, physical activity, independence, understanding, confidence to self-manage and overall impact. When reporting the MSK-HQ all 14 items are summed together (responses coded from 'not at all' = 4 to 'extremely' = 0, except for items 12 and 13 which have the response options in the reverse order) providing a range from 0-56, with higher scores indicating better MSK health status[8]. The EQ-5D-5L questionnaire is a generic quality of life measure, which consists of 5 domains (mobility, self-care, usual activities, pain/discomfort and anxiety/depression) with 5 categorical levels for each domain. In addition, the instrument contains a general health VAS (0-100, 0=best health) item. EQ-5D-5L can be interpreted in a form of an index score or in a form of 243 unique health states. In this study the EQ-5D-5L utility score was calculated using the UK Crosswalk value set, with scores ranging between -0.59 and 1 (full health).[11]. In addition the Orthopaedic Cohorts filled out a joint specific; Oxford Knee Score (OKS), Oxford Hip Score (OHS) or an Oxford Shoulder Score (OSS) as joint specific reference measures. The OKS, OHS and OSS questionnaires follow are patient reported outcome measures with 12-item addressing pain and functional. In each Oxford questionnaire, every item has 5 answer categories in a Likert scale, with items now scored from 0 to 4. The summary score range in all scores is 0 (worst) to 48 (best)[12–14]. The EQ-5D-5L, OKS, OHS and OSS are all widely used PROMs with and we have previously published a detailed analysis of their established measurement properties[15].

This was a longitudinal study and in each group the same scores were recorded at final follow-up (3 months for physiotherapy and 6 months for orthopaedic cohorts) corresponding to the normal clinic practice in each clinical setting. The questionnaires are all patient reported and were filled out by the patients with no assistance from the research or clinical teams.

Evaluation of Responsiveness and Calculation of Minimally Important Change

For peer review only

For each of the prospectively collected cohorts we compared the baseline and follow-up scores (3 months for the Physiotherapy cohort and 6 months for Orthopaedic cohorts). There is no gold standard measurement tool in this context and so we could not apply area under the curve methodology. Following COSMIN guidelines we therefore used the construct approach and we set *a priori* hypotheses of expected positive associations at the moderate or above level between MSK-HQ and the reference measurement tools (EQ-5D-5L, OKS, OHS, OSS) (Table 1). To test these we examined the magnitude and direction of change, calculating the Spearman correlation coefficients (non-parametric data) for comparison of MSK-HQ and other measures, within the different groups. The strength of the positive or negative correlation coefficients were interpreted according to Munro little, if any (0.00–.25), low (.26–.49), moderate (.50–0.69), high (.70–0.89) and very high correlation (.90–1.00).[16] We expected the correlations to be positive and to be moderate or above.

We then performed sub-group analysis calculating and comparing the standardised effect size (SES - the mean change score divided by the standard deviation of the measure at baseline) and the standardised response mean (SRM – the mean change score divided by the standard deviation of the change scores) for the outcome measure used within each cohorts collected.

For the entire cohort the clinically relevant change (improvement or deterioration) was determined by calculating the minimally important change (MIC) using receiver operating characteristic (ROC) curve analysis. For the external anchor we used the following transition item from the Outcome and Experience Questionnaire to identify change in clinical state compared to baseline (Question: 'How would you now rate the problem you recently came to clinic or hospital for?' Response: 'Much better', 'a little better', 'the same', 'a little worse' or 'much worse')[17]. For calculation of the minimally important change the responses were dichotomized using two response groups; (1) improvement = a little better and (2) no improvement = same.

COSMIN guidelines

For peer review only - http://bmjopen.bmj.com/site/about/guidelines.xhtml

We followed COSMIN (COnsensus-based Standards for the selection of health status Measurement INstruments) guidelines for evaluation of responsiveness and calculation of minimally important change[18]. The percentage of missing items is reported. Complete case analyses were performed throughout the analyses for the MSK scores, with no imputation for missing values. The power calculation for sample size of the cohorts has been described in first publication regarding these study groups based on evaluating test-retest properties[8]. Using COSMIN standards the sample size for follow-up data was judged as very good for the total group, the physiotherapy, hip, and knee cohorts (7 times number of items or ≥100 subjects)[19]. The sample size for the shoulder cohort was inadequate.

Statistical analysis

All analyses were conducted in STATA/IC V.14 (StataCorp LP., 2015), SPSS V.22 (IBM Corp, 2013) and Statistical software-R V.3.2.2 (The R Foundation for Statistics, 2015).

Ethical approval

Ethical approval for this study was obtained through the IRAS system (IRAS ref. 168971) via Wales REC 4 committee (REC ref. 15/WA/0040) .

Patient and public involvement

The MSK-HQ questionnaire in a patient reported outcome measure and its development has involved patients in each of the following stages; development of the research question, development of the outcome measures itself and participating in the research.

For peer review only - http://bmjopen.bmj.com/site/about/guidelines.xhtml

Results

Demographic data

There were 592 patients in total who consented to participate in the four cohort studies (210 physiotherapy patients, 150 hip, 150 knee, 82 shoulder). Baseline population characteristics for the overall sample and by each cohort are summarised in Table 2, showing a mean age of 56 years (SD 16.9) and with a split of 56.5% female and 43.5% male. As expected the symptom episode duration was much greater in the secondary care cohorts compared to the community physiotherapy cohort.

Descriptive analysis of baseline and follow-up scores.

Table 3 demonstrates that across the entire cohort there was an improvement in mean baseline score to follow-up score for both MSK-HQ and EQ-5D. A very similar pattern was seen for the physiotherapy cohort. In the joint specific cohorts the same trend occurred across all three scores used in each setting. The response rate for the entire cohort was 70%.

Responsiveness

There was a strong correlation between change scores for MSK-HQ and non-disease specific EQ-5D across the entire cohort with an R value of 0.73 (Table 4). There was moderate correlation between the same measures in the physiotherapy (0.67), knee (0.67) and shoulder cohorts (0.57), with strong correlation in the hip cohort (0.72). When comparing change scores between MSK-HQ and Oxford scores in the joint specific cohorts the correlations for OKS (0.93) and OHS (0.82) were particularly strong, with moderate correlation seen with the OSS (0.61). The correlations meant in all cases the *a priori* hypotheses were accepted (Table 1).

Across all cohorts the MSK-HQ measured a relatively large mean effect size for treatment ranging between 2.14 in the hip cohort and 0.93 in the Physio cohort. Within the Physio cohort greater responsiveness was shown with MSK-HQ compared to EQ-5D, with over twice the effect size measured at 3 months. In the knee cohort MSK-HQ measured a similar effect size to the OKS (1.53 and 1.52 respectively) at 6 months, both of which were greater

For peer review only - http://bmjopen.bmj.com/site/about/guidelines.xhtml

than that calculated using EQ-5D (0.94). A similar pattern was seen within the hip cohort. Within the shoulder cohort the pattern was different with MSK-HQ measuring a greater effect size than OSS and EQ-5D, which delivered similar results across the cohort (both 0.73). The findings were supported by the calculated standardized response mean values (Table 5).

Minimally Important Change

The MIC for MSK-HQ was 5.5 (95% CI 2.7 – 8.3) based on the optimal cut-off value with specificity 0.66 and sensitivity 0.65, with area under the ROC curve 0.66 (See Figure 1).

For peer review only

Discussion

The results of this study demonstrate that the MSK-HQ PROM is responsive to change across patients with a range of musculoskeletal conditions. When considering change scores across the entire cohort the MSK-HQ performed well compared to EQ-5D with a strong correlation between the scores. Within the subgroups the MSK-HQ correlated very well with change measured using the Oxford hip, knee and shoulder scores. This was supported by measured effect sizes, particularly for OKS and OHS. For the community physiotherapy group the correlation with EQ-5D was moderate but there was a much larger measured effect size using the MSH-HQ. The trend for the new score to measure a larger effect size when compared to EQ-5D was seen across all the cohorts.

The requirement to integrate PROMs into clinical management and outcome based commissioning in primary and intermediate care has expanded[20]. The MSK-HQ PROM was designed to be applicable across MSK conditions and to be meaningful to patients particularly in the primary and intermediate care setting[8]. Therefore it is important to reflect on the results of the MSK-HQ across the whole mixed cohort and the community physiotherapy cohort, which does represent a truly mixed population of different MSK conditions. Previous work has shown that the MSK-HQ has excellent test-retest reliability and strong convergent validity with reference standards [8]. Results from the entire cohort and the physiotherapy cohort suggest that the MSK-HQ is responsive to change in the general MSK population and is applicable to the primary and intermediate care setting. Considering its use in this setting we have calculated a single minimally important change (MIC) value for the MSK-HQ for application across all MSK conditions. The MIC value represents the smallest change in MSK-HQ score that represents a meaningful change in symptoms over time for an individual patient within a care pathway, a score that has been calculated for many other outcome measures.[21] Physiotherapy has a very important role to play in the initial non-operative management of nearly all MSK conditions and for many years the outcome measure of choice in this clinical space has been EQ-5D [22–27]. A key vision of the MSK-HQ was to fill the current gap for a single broad health status measure instead of relying on generic health tools such as the EQ-5D-5L which have been shown to be less sensitive to change in MSK populations[9,10]. One key requirement for the MSK-HQ yet to be tested is whether it is more sensitive to change than the EQ-5D-5L. Encouragingly

For peer review only - http://bmjopen.bmj.com/site/about/guidelines.xhtml

he MSK-HQ performed favorably in comparison to EQ-5D in terms of responsiveness to change across the cohort.

Despite the MSH-HQ not being joint or condition specific the new score performed well in responsiveness when compared to the Oxford hip, knee and shoulder scores. In particular the score appears to measure change in response to hip and knee arthroplasty in a similar manner to the OKS and OHS with very high measured correlation coefficients and similar effect sizes. The follow-up the sample size for the shoulder cohort was inadequate and further work is required to determine responsiveness in this group of patients.

Our study does have a number of limitations that must be addressed. We have not collected any information regarding detailed nature of pathology or treatment within each cohort, such as stage of disease, time to treatment or detail of reconstructive surgery. Our aim was not to explore these issues but only to measure change in the MSK-HQ before and after treatment to calculate responsiveness across the whole MSK cohort. Our study does have missing data from the follow-up cohort. However overall the effective response rate for the entire cohort was 70%, which is considered acceptable[28]. In calculating responsiveness the sample size for the entire group and all individual cohorts, other than shoulder, was very good as judged against COSMIN standards. Although the study was performed solely in the NHS and the study population did not cover all MSK diagnoses, we believe the study group is representative and the results generalisable to the broad group of MSK patients who exist in all-healthcare systems.[8]

In the our previous publication publication regarding the production of MSK-HQ we laid out a set of prerequisites that should be met for its creation[8]. We believe that our previous findings together with the results of this study show that these conditions have now been met. The score has been coproduced with patients and clinicians and reflects aspects of health that were meaningful to both. It can provided an MSK specific quantification of a person's musculoskeletal health. The score can be used across the MSK pathway with different MSK conditions and treatment targets. It does demonstrate robust psychometric properties and it is responsive to change enabling longitudinal measurement and the monitoring of changes over time. In this study we have demonstrated that it is applicable

For peer review only - http://bmjopen.bmj.com/site/about/guidelines.xhtml

for use by different MSK health professionals and feasible for use in routine clinical practice across the MSK patient pathway.

The expansion of MSK intermediate care in within the NHS has highlighted the need for a generic PROM to assist in developing outcome based commissioning of care. The measurement property profile of the MSK-HQ PROM appears to support its use in this area, although its role is still evolving. Increasingly there is a common clinical pathway for most MSK patients within the NHS. Patients initially seek advice and treatment from their GP and if their condition does not settle are referred to a musculoskeletal intermediate care referral hub, which acts as a gateway to secondary care. The pathway is not joint or condition specific so a generic MSK PROM to measure clinical change and response to treatment is required for the process. The MSK-HQ appears to demonstrate the appropriate measurement properties and responsiveness to fulfill this role.

Conclusion

In summary the findings from this study show that the MSK-HQ appears to be responsive to change across a range of MSK conditions, performing favourably when compared to EQ-5D.

For peer review only

References:

1. Margham T. Musculoskeletal disorders: time for joint action in primary care. Br J Gen Pract. 2010;61(592):657–8.
2. Jordan KP, Kadam UT, Hayward R, Porcheret M, Young C, Croft P. Annual consultation prevalence of regional musculoskeletal problems in primary care : an observational study. 2010;
3. Maddison P, Jones J, Breslin A, Barton C, Fleur J, Lewis R, et al. Quality improvement report Improved access and targeting of musculoskeletal services. 2004;1325–7.
4. Hussenbux A, Morrissey D, Joseph C, Mcclellan CM. Intermediate Care pathways for musculoskeletal conditions – Are they working ? A systematic review. Physiotherapy [Internet]. 2015;101(1):13–24. Available from: http://dx.doi.org/10.1016/j.physio.2014.08.004
5. Jl J, Ma H, Eej M, Ne F. Interventions to improve adherence to exercise for chronic musculoskeletal pain in adults (Review). 2010;(1).
6. Roberts C, Dolman EA, Adebajo AO, Underwood M. A national qualitative survey of community- based musculoskeletal services in the UK. 2003;(May):1074–8.
7. DOH. The Musculoskeletal Services Framework A joint responsibility : doing it differently. [Internet]. 2006 [cited 2018 Apr 14]. Available from: www.dh.gov.uk/publications
8. Hill JC, Kang S, Benedetto E, Myers H, Blackburn S, Smith S, et al. Development and initial cohort validation of the Arthritis Research UK Musculoskeletal Health Questionnaire (MSK-HQ) for use across musculoskeletal care pathways. BMJ Open. 2016;6(8):1–10.
9. Whynes DK, McCahon RA, Ravenscroft A, Hodgkinson V, Evley R, Hardman JG. Responsiveness of the EQ-5D health-related quality-of-life instrument in assessing low back pain. Value Heal [Internet]. 2013;16(1):124–32. Available from: http://dx.doi.org/10.1016/j.jval.2012.09.003
10. Tordrup D, Mossman J, Kanavos P. Responsiveness of the EQ-5D to clinical change: Is the patient experience adequately represented? Int J Technol Assess Health Care. 2014;30(1):10–9.
11. Lloyd MHCGA. Development and preliminary testing of the new five-level version

For peer review only

For peer review only - http://bmjopen.bmj.com/site/about/guidelines.xhtml

of EQ-5D (EQ-5D-5L). 2011;1727–36.

12. Dawson J, Fitzpatrick R, Carr A. Questionnaire on the perceptions of patients about shoulder surgery. J Bone Joint Surg Br [Internet]. 1996;78(4):593–600. Available from: http://www.ncbi.nlm.nih.gov/pubmed/8682827

13. Dawson J, Fitzpatrick R, Carr A, Murray D. Questionnaire on the Perceptions of Patients About Total Hip Replacement. J Bone Jt Surg Br [Internet]. 2015;78–B(2):185–90. Available from: http://www.bjj.boneandjoint.org.uk/content/78-B/2/185.full.pdf+html

14. Dawson J, Fitzpatrick R, Murray D, Carr A. Questionnaire on the Perceptions of Patients about Total Knee Replacement. J Bone Joint Surg Br [Internet]. 1998;80(1):63–9. Available from: http://www.ncbi.nlm.nih.gov/entrez/query.fcgi?cmd=Retrieve&db=PubMed&dopt=Citation&list_uids=9460955

15. Harris K, Dawson J, Gibbons E, Lim C, Beard D, Fitzpatrick R, et al. Systematic review of measurement properties of patient-reported outcome measures used in patients undergoing hip and knee arthroplasty. Patient Relat Outcome Meas [Internet]. 2016;Volume 7:101–8. Available from: https://www.dovepress.com/systematic-review-of-measurement-properties-of-patient-reported-outcom-peer-reviewed-article-PROM

16. Munro BH. Statistical methods for health care research. 5th ed. Philadelphia: Lippincott Williams & Wilkins; 2005.

17. Gibbons E, Hewitson P, Morley D, Jenkinson C, Fitzpatrick R. The Outcomes and Experiences Questionnaire: development and validation. Patient Relat Outcome Meas [Internet]. 2015;6:179–89. Available from: http://www.ncbi.nlm.nih.gov/pubmed/26213480%5Cnhttp://www.pubmedcentral.nih.gov/articlerender.fcgi?artid=PMC4509457

18. Mokkink LB, Terwee CB, Patrick DL, Alonso J, Stratford PW, Knol DL, et al. The COSMIN study reached international consensus on taxonomy, terminology, and definitions of measurement properties for health-related patient-reported outcomes. J Clin Epidemiol. 2010;63(7):737–45.

19. Mokkink LB, Prinsen CA, Alonson J, Bouter LM, de Vet HCW, Terwee CB. COSMIN methodology for systematic reviews of Patient-Reported Outcome Measures

For peer review only

(PROMs). COSMIN User Manual. 2017.

20. The Musculoskeletal Services Framework A joint responsibility : doing it differently.

21. Beard DJ, Harris K, Dawson J, Doll H, Murray DW, Carr AJ, et al. Meaningful changes for the Oxford hip and knee scores after joint replacement surgery. J Clin Epidemiol. 2015;68(1):73–9.

22. Desmeules F, Roy J-S, MacDermid JC, Champagne F, Hinse O, Woodhouse LJ. Advanced practice physiotherapy in patients with musculoskeletal disorders: a systematic review. BMC Musculoskelet Disord [Internet]. 2012;13(1):107. Available from: http://bmcmusculoskeletdisord.biomedcentral.com/articles/10.1186/1471-2474-13-107

23. Holdsworth L, Webster V, McFayden A. What are the costs to NHS Scotland of self-referral to physiotherapy? Physiotherapy. 2007;93:3–11.

24. Holdsworth L, Webster V, McFayden A. What are the costs to NHS Scotland of self-referral to physiotherapy? Physiotherapy. 2007;

25. McGill T. Effectiveness of physical therapists servicing as primary care musculoskeletal providers as compared to family practice providers in a deployed combat location: a retrospective medical chart review. Mil Med. 2013;178(10):115–1120.

26. Fritz JM, Cleland JA, Brennan GP. Does adherence to the guideline recommendations for active treatments improve quality of care for patients with acute low back pain delivered by physical therapists? Med Care [Internet]. 2007;45(10):973–80. Available from: http://search.proquest.com/docview/621889514?accountid=9941

27. Childs JD, Whitman JM, Sizer PS, Pugia ML, Flynn TW, Delitto A. A description of physical therapists' knowledge in managing musculoskeletal conditions. BMC Musculoskelet Disord. 2005;6.

28. Fincham JE. Response rates and responsiveness for surveys, standards, and the Journal. Am J Pharm Educ. 2008;72(2):43.

For peer review only

Figure Legends

Figure 1:

A graph showing the receiver operating characteristic (ROC) analysis using MSK-HQ and EQ-5D Index change scores across the entire cohort.

For peer review only

For peer review only - http://bmjopen.bmj.com/site/about/guidelines.xhtml

For peer review only

Number	Hypotheses	Result
1	The correlation between the MSK-HQ and EQ-5D within the entire cohort is moderate (r ≥ 0.50) or above	Accepted
2	The correlation between the MSK-HQ and EQ-5D within the physiotherapy cohort is moderate (r ≥ 0.50) or above	Accepted
3	The correlation between the MSK-HQ and EQ-5D within the knee cohort is moderate (r ≥ 0.50) or above	Accepted
4	The correlation between the MSK-HQ and EQ-5D within the hip cohort is moderate (r ≥ 0.50) or above	Accepted
5	The correlation between the MSK-HQ and EQ-5D within the shoulder cohort is moderate (r ≥ 0.50) or above	Accepted
6	The correlation between the MSK-HQ and OKS within the knee cohort is moderate (r ≥ 0.50) or above	Accepted
7	The correlation between the MSK-HQ and OHS within the hip cohort is moderate (r ≥ 0.50) or above	Accepted
8	The correlation between the MSK-HQ and OSS within the knee cohort is moderate (r ≥ 0.50) or above	Accepted

MSK-HQ: Musculoskeletal Healthcare Questionnaire, EQ-5D-5L: , OKS: Oxford Knee Score, OHS: Oxford Hip Score, OSS: Oxford Shoulder Score, r = Spearman Correlation Coefficient.

Table 1: A table demonstrating the eight *a-priori* hypotheses that were examined

For peer review only

**Patient characteristics**	***All participants***	***Physio***	***Hip***	***Knee***	***Shoulder***
**Number**	592	210	150	150	82
**Age (years),** ***mean (SD)***	56.0 *(16.9)*	53.3 *(15.5)*	55.6 *(17.2)*	65.7 *(13.8)*	53.0 *(16.7)*
**Female,** ***n (%)***	326 *(55.1)*	112 *(53.3)*	88 *(58.7)*	89 *(59.3)*	37 *(45.1)*
**Male,** ***n (%)***	266 *(43.5)*	98 *(46.7)*	62 *(41.3)*	61 *(40.7)*	45 *(54.9)*

Table 2: A table showing demographic characteristics of patients recruited across the four cohorts.

For peer review only

Cohort	Surveys for each Cohort [Lower-Upper range}	Baseline Mean	Baseline SD	Follow-up* Mean	Follow-up* SD	Follow-up Response rate (%)
**Total** (n=592)	MSK-HQ [0,56]	28.6	9.6	41.3	11.4	70
	EQ-5D-5L [-0.59,1]	0.5	0.3	0.7	0.2	
**Physio** (n=210)	MSK-HQ	30.5	9.6	40.0	11.3	63
	EQ-5D-5L [-0.59,1]	0.6	0.3	0.7	0.2	
**Hip** (n=150)	MSK-HQ	24.9	8.3	43.0	12.0	71
	EQ-5D-5L [-0.59,1]	0.4	0.2	0.7	0.3	
	OHS [0,48]	20.4	8.6	37.4	10.0	
**Knee** (n=150)	MSK-HQ	27.5	9.0	40.8	11.5	82
	EQ-5D-5L [-0.59,1]	0.5	0.3	0.7	0.2	
	OKS [0,48]	20.9	8.8	34.6	10.1	
**Shoulder** (n=82)	MSK-HQ	32.1	10.4	42.9	9.4	44
	EQ-5D-5L [-0.59,1]	0.6	0.3	0.7	0.2	
	OSS [0,48]	29.6	10.3	37.5	7.4	

Missing data for baseline MSK-HQ: all participants n=50 (8.2%), Physiotherapy cohort n=5 (2.4%), hip cohort n=4 (2.7%), knee cohort n=22 (14.7%), shoulder cohort n=2 (2.4%).

**Follow-up time point: Physiotherapy cohort = 3 months, hip/knee/shoulder cohorts = 6 months.*

MSK-HQ: Musculoskeletal Healthcare Questionnaire, EQ-5D-5L: The European Quality of Life Questionnaire (range -0.59 and 1), OKS: Oxford Knee Score (range 0-48), OHS: Oxford Hip score (range 0-48) , OSS: Oxford Shoulder Score (range 0-48).

Table 3: A table showing base line and follow-up outcomes scores for each cohort.

For peer review only - http://bmjopen.bmj.com/site/about/guidelines.xhtml

For peer review only

Instrument	Comparator	Cohort	Spearman Correlation Coefficient [r]
**MSK-HQ**	***EQ-5D***	*Total*	0.73
**MSK-HQ**	***EQ-5D***	*Physio*	0.67
		Hip	0.77
		Knee	0.68
		Shoulder	0.60
**MSK-HQ**	***OHS***	*Hip*	0.87
	OKS	*Knee*	0.92
	OSS	*Shoulder*	0.77

Table 4: A table displaying correlations in change score between MSK-HQ and other reference outcome scores (EQ-5D, OHS OKS and OSS) in the entire group and the cohorts (physiotherapy, hip, knee and shoulder)

For peer review only

Cohort *(Number responding)*		Effect size (95% CIs) [1]	SRM (95% CIs) [2]
**Physio** *(n=147)*	MSK-HQ	0.93 (0.66,1.20)	0.99 (0.71,1.26)
	EQ-5D-5L	0.43 (0.18,0.68)	0.46 (0.21,0.71)
**Knee** *(n=107)*	MSK-HQ	1.53 (1.18,1.86)	1.05 (0.74,1.35)
	OKS	1.52 (1.19,1.85)	1.26 (0.95,1.57)
	EQ-5D-5L	0.94 (0.65,1.23)	0.87 (0.58,1.15)
**Hip** *(n=123)*	MSK-HQ	2.14 (1.73,2.53)	1.56 (1.21,1.90)
	OHS	1.93 (1.53,2.33)	1.59 (1.22,1.95)
	EQ-5D-5L	1.21 (0.88,1.52)	1.03 (0.72,1.34)
**Shoulder** *(n=36)*	MSK-HQ	1.05 (0.52,1.57)	1.08 (0.55,1.60)
	OSS	0.73 (0.22,1.23)	0.99 (0.45,1.51)
	EQ-5D-5L	0.73 (0.24,1.20)	0.76 (0.29,1.24)

1. Using baseline (BL) standard deviation (SD) 2. Using paired differences standard deviation (SD)

Table 5: A table displaying effect size and standard response mean for MSK-HQ and other established outcome score in the 4 cohorts (physiotherapy, hip, knee and shoulder).

For peer review only

Acknowledgements

The authors thank the clinical teams and patients that participated in the study. The authors acknowledge support from the Oxford NIHR Biomedical Research Centre.

Contributors

AP, RF, BE, DB, EH, JG, JCH contributed to the conception and design of the work.

JCH, EH, SS, SK, EB, JS, KB, DB, JR, SG-J and AP were involved in running the cohorts and collecting data.

RO, AP, SK and JCH were involved in the analyses and AP, JCH and RF in the interpretation of the data.

AP, RO, EH, KB, SS, JS, JG, JR, SG-J, DB, EB, RF, JH, BE were involved in the drafting of the manuscript and its revision for important intellectual content, and gave final approval for the manuscript.

Funding

This project was funded by Arthritis Research UK (Ref. 20518). Professor Andrew Price is part funded through the Oxford NIHR Biomedical Research Centre.

Competing interests

None declared.

Patient consent

All participants provided informed written consent.

Ethics approval

Ethical approval for this study was obtained through the IRAS system (IRAS ref. 168971) via Wales REC 4 committee (REC ref. 15/WA/0040) .

Data sharing statement

Additional data can be accessed on request to the corresponding author: andrew.price@ndorms.ox.ac.uk

For peer review only

For peer review only

For peer review only - http://bmjopen.bmj.com/site/about/guidelines.xhtml

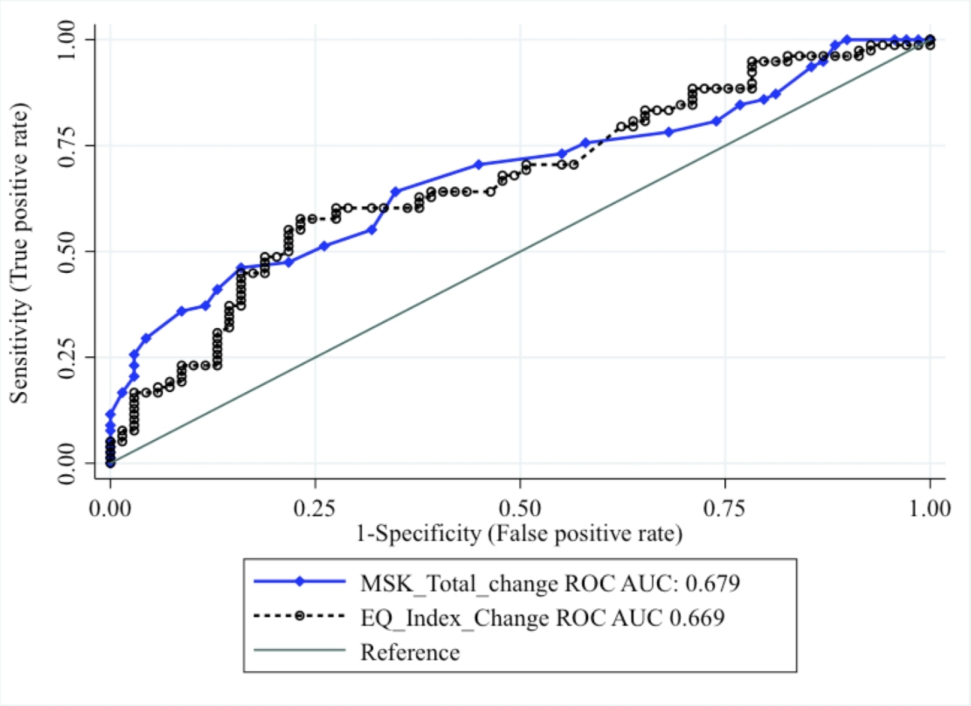

A graph showing the receiver operating characteristic (ROC) analysis using MSK-HQ and EQ-5D Index change scores across the entire cohort.

297x209mm (300 x 300 DPI)
